# Excitatory nicotinic signaling drives action potential bursting in dopaminergic axons

**DOI:** 10.64898/2025.12.19.695584

**Published:** 2025-12-21

**Authors:** Paul F. Kramer, Anthony Yanez, Faye Clever, Renshu Zhang, Zayd M. Khaliq

**Affiliations:** 1 Cellular Neurophysiology Section, National Institute of Neurological Disorders and Stroke, National Institutes of Health, Bethesda, MD, 20892, USA; 2 Aligning Science Across Parkinson’s (ASAP) Collaborative Research Network, Chevy Chase, MD 20815, USA; 3 Present address: Department of Molecular, Cellular, and Developmental Biology, University of Michigan, Ann Arbor, MI, 48109, USA; 4 Present address: Biochemistry, Cellular and Molecular Biology Graduate Program, Johns Hopkins School of Medicine, Baltimore, MD, 21287, USA; 5 Present address: Department of Molecular & Cellular Biology, University of California, Berkeley, Berkeley, CA, 94720, USA

## Abstract

Axons of dopamine (DA) neurons express nicotinic receptors (nAChRs) that have been shown to both facilitate and suppress striatal DA release, but the mechanisms underlying these opposing actions are unclear. We combined axonal recordings and calcium imaging approaches to examine the effects of nAChRs on DAergic axon excitability. Activation of nAChRs consistently depolarized DAergic axons and increased the probability of evoking action potentials. Calcium imaging experiments showed that weak or moderate stimulation of striatal cholinergic interneurons (CINs) activated DAergic axons, while strong stimulation blunted multipulse protocols. Axon recordings showed that highly synchronous stimulation of CINs triggered a rapid (~125 Hz) burst of 2–4 action potentials in DAergic axons. Imaging experiments showed that nAChR-evoked axon bursts led to prolonged refractoriness, which was mimicked by direct burst stimulation. Thus, nAChR activation locally enhances excitability of DAergic axons over a range of intensities, including the production of locally generated burst firing and subsequent refractory inhibition.

## INTRODUCTION

Dopaminergic neurons of the ventral midbrain (DANs) regulate behaviors such as motivation, reward learning, and saliency through striatal dopamine (DA) release (Berke, 2018; Costa & Schoenbaum, 2022; Gershman et al., 2024; Wise & Jordan, 2021). Signaling in DANs results from the interaction of synaptic inputs in the soma and dendrites that regulate the generation of action potentials (APs) at the axon initial segment (Hausser et al., 1995). These APs propagate to terminals triggering DA release, thus relaying somatodendritic activity. In addition to this standard view of neuronal signaling, DAergic axons express receptors that are positioned to locally modulate striatal DA release in a manner that is distinct from somatodendritic signaling (Seddon & Kramer, 2025). Local receptor modulation of DAergic axonal excitability represents a distinct strategy to shape signaling from DANs onto targets within the striatum.

One receptor expressed by the axons of DANs (DAergic axons) is the nicotinic acetylcholine receptor (nAChR), activated by transmission from striatal cholinergic interneurons (CINs) (Brimblecombe et al., 2018). Overall, the effects of axonal nAChRs on DA signaling are still unclear, with both positive and negative modulation of terminal DA release described. There is evidence that nAChR-mediated DA release is involved in movement and motivation in mice (Liu et al., 2022; Mohebi et al., 2019) and learning in song birds (Qi et al., 2025). Other evidence suggests no causal relationship in mice (Azcorra et al., 2023; Chantranupong et al., 2023; Krok et al., 2023), or perhaps an opposing role where nAChRs inhibit DA release (Zhang et al., 2025). These data highlight the need for better mechanistic understanding at the cellular level.

Physiological studies have highlighted both excitatory and inhibitory roles for axonal nAChRs in modulating dopamine release, but the mechanisms remain unresolved. Optical or spontaneous activation of striatal CINs robustly drives nAChR-mediated DA release in both dorsal and ventral striatal regions (Cachope et al., 2012; Matityahu et al., 2023; Threlfell et al., 2012; Yorgason et al., 2017; Zhou et al., 2001). The mechanism driving CIN-mediated DA release is nAChR-mediated initiation of APs in the DAergic axon (Liu et al., 2022) through a novel form of neurotransmission between axons (Kramer et al., 2022). Conversely, early studies suggested an additional role of nAChRs in inhibiting DA release. Activation of nAChRs potentiated DA release for bursts of stimulations at low-frequencies (<20 Hz), but suppressed DA release following high-frequency (>20 Hz) stimulations (Rice & Cragg, 2004; Zhang & Sulzer, 2004), suggesting that axonal nAChRs function as low-pass filters that amplify low-frequency DA release but dampen DA release at high-frequencies. It remains unclear how high-frequency stimulation could be inhibited by nAChR signaling in DAergic axons.

Here, we examined how nAChRs bidirectionally modulate the excitability of DAergic axons using a combination of patch-clamp voltage measurements and calcium imaging from DAergic axons in the dorsal-medial striatum (DMS). We demonstrate that activation of axonal nAChRs consistently produced subthreshold depolarization and AP firing, confirming their role as excitatory receptors. Calcium imaging experiments examining coincident activation of CINs and DAergic axons showed that sub-maximal activation of CINs enhanced DAergic axonal excitability while strong stimulation could lead to inhibition of axonal DAergic signals. Axonal patch-clamp experiments showed that a single electrical shock or light pulse used to activate CINs resulted in a nAChR-dependent high-frequency burst of APs in DAergic axons followed by a prolonged state of reduced excitability that lasted 60–80 ms. Thus, we reveal a timing dependency to CIN modulation of DAergic axonal excitability. Strong excitation from striatal CINs leads to a novel form of rapid axonal bursting, but which also produces a prolonged period of refractory inhibition

## RESULTS

### Quantifying nAChR modulation of bursting in DAergic axons by imaging jGCaMP8f

**Figure 1.**
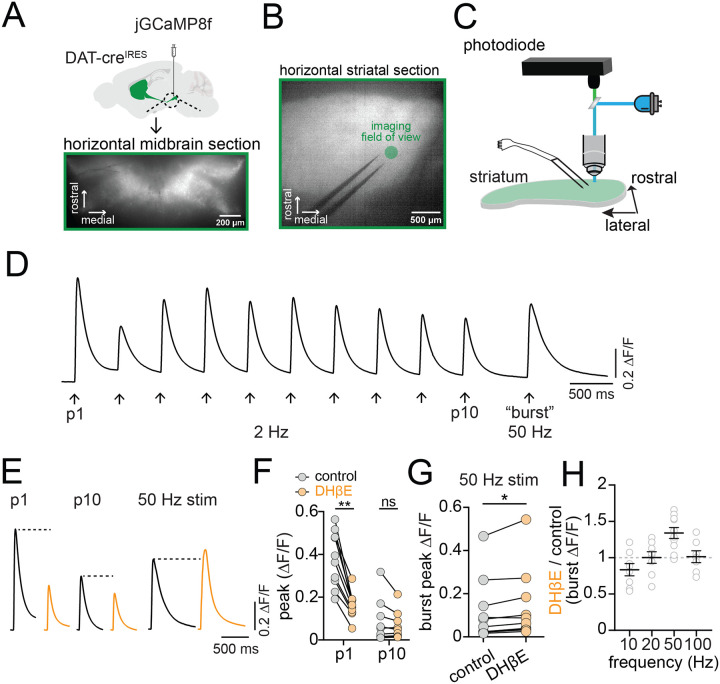
nAChRs inhibit burst-mediated excitability of DAergic axons **A.** Cre-dependent jGCaMP8f AAV injected into the ventral midbrain of DAT-cre mice; lower panel shows infected neurons. **B.** Fluorescent image of horizontal DMS section with jGCaMP8f expression, bipolar electrode, and imaging site (green circle). **C.** Cartoon of experimental setup: LED excitation and photodiode recording of jGCaMP8f fluorescence. **D.** Example photodiode trace showing jGCaMP8f responses to repeated DMS electrical stimulation with 10 stimuli at 2Hz followed by 5 stimuli at 50 Hz. **E.** Example jGCaMP8f traces showing first (P1), last (P10) in 2 Hz train, and 50 Hz burst stimulation in control (black) and DHβE (orange) ACSF. **F.** Collected peak jGCaMP8f signal amplitudes in control (gray symbols) and DHβE (orange symbols) for P1 and P10. **G.** Collected peak signal amplitudes for 50 Hz burst stimulation in control solutions versus DHβE. **H.** Ratio of jGCaMP8f signal amplitudes measured in DHβE over control aCSF for 10, 20, 50 and 100 Hz stimulation frequencies. *p<0.05, **p<0.01, ns > 0.05. All experiments in D2R, GABA-B, and mAChR antagonists.

Nicotinic receptors (nAChRs) expressed on the axons of DANs have been suggested to perform a filtering action on striatal DA release, enhancing DA release during single and low frequency stimulations, and inhibiting DA release evoked by burst stimulations (Rice & Cragg, 2004; Zhang & Sulzer, 2004). The influence of nAChRs on striatal DA has been primarily examined using carbon fiber voltametric measurements (Adrover et al., 2020; Stouffer et al., 2015; Threlfell et al., 2012; Zhou et al., 2003), which quantify the concentration of DA released from thousands of vesicle release sites (Robinson et al., 2003). However, transmitter release is largely determined by processes upstream of vesicular release, like the frequency and characteristics of axonal AP firing. Whether nAChR-mediated filtering results from effects on axonal excitability is unclear. We tested the effect of nAChR signaling on DAergic axonal excitability using electrical stimulation of the DMS in acutely prepared brain sections. This results in co-stimulation of DAergic axons by two temporally separated processes: immediate direct excitation from the electrical stimulation and delayed synaptic excitation through acetylcholine (ACh) release from CIN terminals onto axonal nAChRs. We monitored calcium (Ca2+) influx in striatal DAergic axons by expressing the genetically-encoded indicator jGCaMP8f (Zhang et al., 2023) in DANs of the SNc (Figure 1A–C). Tonic and high-frequency bursting are characteristic patterns of DANs (Grace & Bunney, 1984). Therefore, we mimicked this activity by stimulating trains of 10 pulses at 2 Hz to produce a tonic pattern of activity, followed by a 750 ms pause and then a burst stimulation of 5 pulses at 50 Hz (Figure 1D).

To examine the contribution of nAChR signaling to DAergic axon excitability, we tested the effect of the nAChR antagonist dihydro-β-erythroidine hydrobromide (DHβE, 1 μM) on stimulated DAergic axon signals. We found that the amplitude of the first pulse (P1) in the tonic stimulation train was significantly reduced following DHβE inhibition of nAChRs (avg ΔF/F, control=0.38; DHβE=0.16, t(18)=9.7, p<0.0001; Figure 1E and 1F), but the tenth pulse (P10) amplitude was unaffected (avg ΔF/F, control=0.08; DHβE=0.06, t(18)=0.77, p=0.45). By contrast, 50 Hz burst stimulation resulted in a Ca2+ signal that was significantly increased in amplitude following DHβE inhibition of nAChRs (avg burst peak ΔF/F, control=0.12, DHβE=0.14, t(9)=2.95, p=0.016; Figure 1G). In separate experiments, we tested the effect of DHβE on Ca2+ signals following 5 pulse stimulations delivered at 10, 20 and 100 Hz (Figure 1H). Plots of the ratio of Ca2+ signals in DHβE relative to control showed that nAChR inhibition reduced the amplitude of 10 Hz stimulation signals, enhanced the 50 Hz signals and had no effect on signals evoked by 20 Hz and 100 Hz (avg DHβE/control Ca2+ signal, 10 Hz, 0.83 +/− 0.08; 20 Hz, 1.00+/−0.08; 100 Hz, 1.01+/−0.08). Therefore, these results demonstrate that nAChRs enhance DAergic axon excitability during CIN/DAN axon co-stimulation at single or low-frequencies below 20 Hz. However, for higher frequency stimulation at 50 Hz, application of nAChR antagonists led to a significant increase in DAergic axon Ca2+ signals suggesting they play a complicated, but net inhibitory, role in regulating DAergic axon excitability following high-frequency burst stimulation.

### Action potential bursting in distal DAergic axons

**Figure 2:**
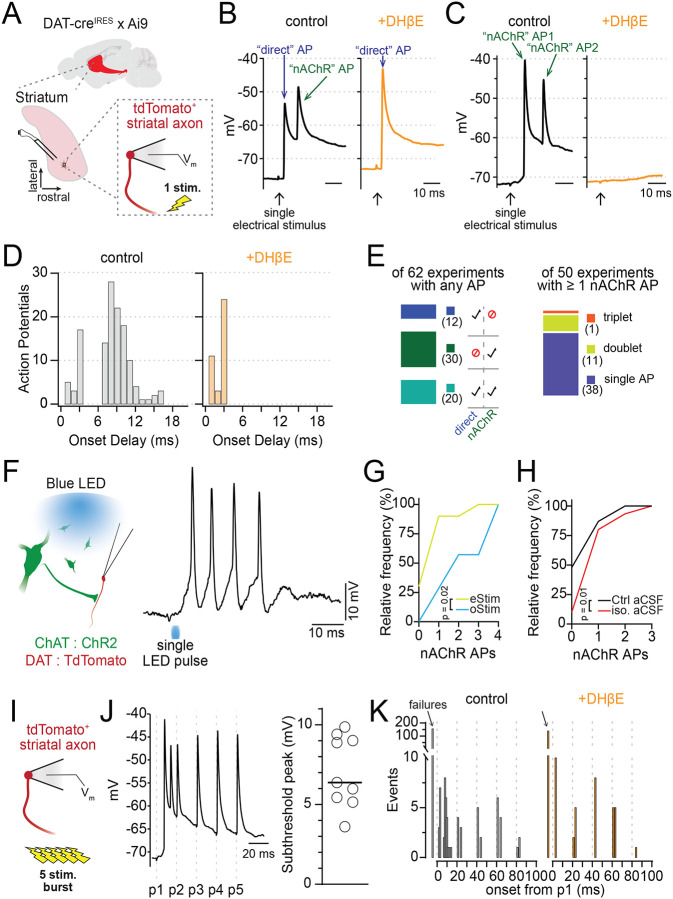
Axonal nAChR signaling initiates action potential bursting. **A.** Diagram of perforated patch recordings from DAT+ axons. **B-C.** Example traces of two AP burst patterns evoked by single electrical stimuli: direct AP with a nAChR-dependent AP (B), or two nAChR-dependent APs (C). DHβE (1 μM) is a nAChR antagonist. **D.** Frequency distribution of AP onset times in control (left) and DHβE (orange). **E.** (Left) Number of axons recording experiments (n= 62 total) with direct, nAChR-evoked, or both AP types; (Right) subset showing cases with 1–3 nAChR-mediated APs per experiment. **F.** (Left) ChR2 and TdTomato expression in ChAT+ and DAT+ neurons, respectively, via AAV in ChAT-cre × DAT-cre mice. (Right) Example of AP bursts in a DAergic axon following a single blue light pulse in isolation aCSF. **G.** Cumulative frequency: number of experiments with 1–4 APs, compared between electrical and optical stimulation. **H.** Cumulative frequency: number of experiments with 1–3 APs, compared for electrical stimulus experiments in control and isolation aCSF. **I.** Experiment diagram for burst stimulation of DAergic axons. **J.** Example DAergic axon voltage trace during 5-stim, 50 Hz bursts in iso. aCSF and collected peak depolarization of the subthreshold burst envelope. **K.** Frequency distribution of AP onset times during 50 Hz bursts in isolation aCSF (black) and DHβE (orange). Iso. aCSF contained D2R, GABA-B, and mAChR antagonists.

To determine how nAChRs enhance and inhibit DAergic axon excitability at low and high frequencies, respectively, we next recorded the membrane potential of DAergic axons in the DMS following single or burst stimulation protocols. We found that a single pulse of electrical stimulation could evoke an AP in the DAergic axon as expected. Surprisingly, we also observed that a single stimulus resulted in a high-frequency burst of 2–3 axonal APs (Figure 2A–2C; Supplemental Figure 1A,B). In trials with two or more APs, the first AP occurred 1.74 ± 0.84 ms after the electrical pulse, and the second AP occurred an average of 9.22 ± 1.36 ms after the stimulus (median instantaneous rate, 122 ± 26.2 Hz, n=16). Importantly, we found that all APs that occurred later than 6 ms following the stimulus were abolished by DHβE, while APs that occur earlier were typically unaffected (Figure 2D). Of the 50 trials we analyzed where nAChR-mediated APs were evoked, we saw a single AP in 38 trials, a doublet in 11 trials, and a triplet in 1 trial (Figure 2E). Based on these observations, we concluded that earliest APs resulted from direct excitation of the DAergic axon (“direct”), while delayed APs (> 6ms delay from stimulation) were likely evoked by activation of nAChRs.

Occasionally the first AP of the burst was delayed, occurring 9.51 ± 1.54 ms after the stimulation, with the second nAChR-evoked AP at 17.5 ± 2.5 ms. In one case, a third nAChR-evoked AP occurred 20.4 ms following the single electrical pulse. In these cases, the burst frequency was not significantly different from bursts starting with early direct APs (median instantaneous rate, 125 ± 42.8 Hz, p=0.35, n=12; Supplemental Figure 1B). Of note, we also recorded a single example of a spontaneously occurring high frequency burst of APs (Supplemental Figure 1E), indicating that nAChR-mediated AP bursting in DAergic axons are not dependent on external stimulation.

To test the involvement of CINs in nAChR-mediated bursting in DAergic axons, we expressed channelrhodopsin (ChR2) in striatal CINs to selectively evoke ACh release these neurons while recording the membrane voltage from nearby DAergic axons (Figure 2F). Similarly, we found that single light pulses evoked robust, high-frequency bursts of 2–4 APs in DAergic axons (Figure 2F; Supplemental Figure 1C–D). Comparisons of electrical and optical stimulation showed that optical stimulation evoked a bursting at a higher probability and evoked bursts with greater numbers of APs on average (Figure 2G; D=0.59, p=0.02, n(eStim) = 10, n(oStim) = 7).

The striatum is a dense hub of local interneurons and long-range inputs, many of which alter both the activity of CINs and the terminal release of DA (Burke et al., 2017; Liu & Kaeser, 2019; Ratna & Francis, 2025; Sulzer et al., 2016). Central to the axo-axonic transmission of ACh from CINs to DAergic axons are the modulatory neurotransmitters DA, GABA and ACh. To investigate if inhibition from these transmitters alters the initiation of APs in DAergic axons, we compared the frequency of nAChR-evoked APs in the presence of inhibitors against muscarinic-, D2-, and GABAb-receptor (“iso. aCSF”, see methods). We observed a significant increase in multispike bursts when recordings were made in iso. aCSF. (p=0.01, control n=46, iso. aCSF n=30; Figure 2H), suggesting that inhibitory GPCRs limit, but do not prohibit, nAChR-dependent bursting in DAergic axons. GABAa receptors can modulate the integration of nAChR activity in DAergic axons, but they do so equally for single- or burst-stimulated activity (Brill-Weil et al., 2025).

To determine whether a train of electrical stimulation could evoke more nAChR-mediated spiking, we next evoked a 5-pulse train at 50 Hz with the bipolar electrode while recording voltage from DAergic axons of the DMS (Figure 2I). As earlier, we observed a burst of nAChR-mediated APs following the first stimulation and directly stimulated APs in the DAergic axons, as well as a broad depolarization envelope of 7.08 +/− 2.24 mV (Figure 2J). Having already established the distinction between direct and nAChR-mediated APs in DAergic axons, we subsequently used the timing of these events to classify them. Action potentials with an onset before 3 ms following an electrical stimulation were classified as direct APs, while those with an onset between 7 and 20 ms after an electrical stimulation were classified as nAChR-mediated APs. Using this classification, we observed that nAChR-mediated APs only occurred after the first electrical pulse in the 5-pulse burst (Figure 2K). Pulses 2 through 5 of the train were made up only of direct APs. Supporting this conclusion, the application of DHβE to block nAChR signaling eliminated only those APs with an onset between 7 and 20 ms after the first electrical stimulation (Figure 2K).

In summary, we find that single electrical or optical stimulations of ACh release in brain tissue can evoke AP bursting in DAergic axons at very high instantaneous frequencies. Furthermore, we show that optical stimulation of CIN is significantly more effective in driving axonal bursting in DAergic axons relative to electrical stimulation.

### Phasic subthreshold depolarization in DAergic axons is excitatory

**Figure 3:**
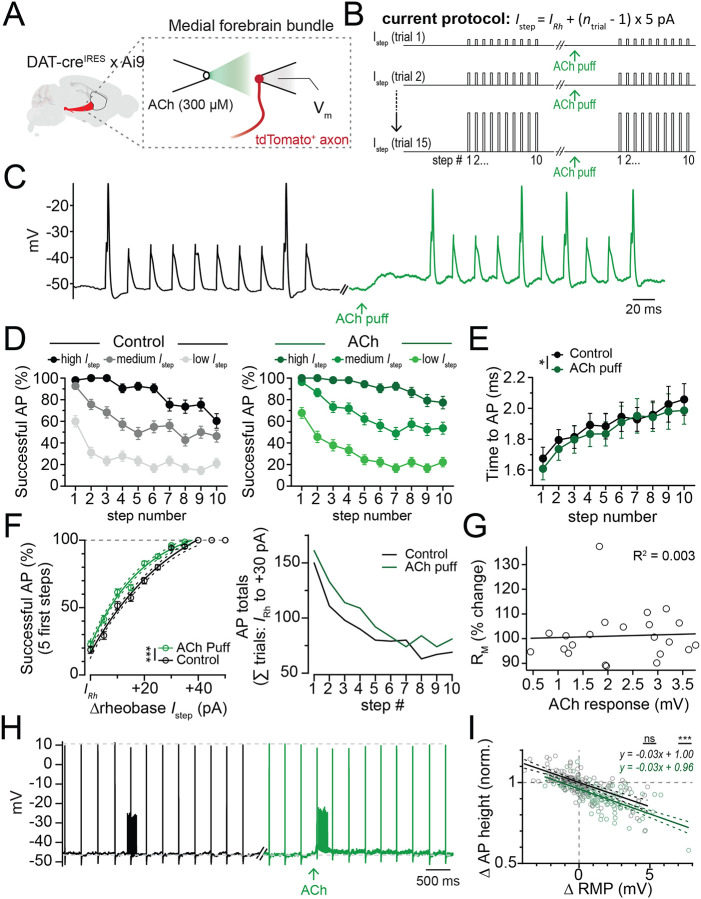
Subthreshold nAChR depolarizations are excitatory. **A.** Experimental setup: DAergic axons labeled via DAT-cre × Ai9 mice; perforated patch recordings from MFB axons; nAChRs activated by local ACh ejection. **B.** Current injection protocol to assess AP initiation probability; trial 1 current (*I*_Rh_) near rheobase. **C.** Example DAergic axon traces during 10-pulse, 50 Hz current trains in control (black) and after ACh pressure ejection (green). **D.** Current injections grouped by amplitude: low, medium, high for control (black) and ACh pressure ejection (green). **E.** Action potential latency per pulse in control and during ACh ejection. **F.** (Left) Percentage of APs generated within the first 5 steps of a 10-step burst for varying current levels near rheobase (X); within-trial comparison in control (black) and ACh ejection (green). (Right) Total APs summed across all current injection levels from 15 axons, compared within trials (control vs. ACh ejection) for current step amplitudes from *I*_Rh_ to *I*_Rh_ + 30 pA. **G.** Change in input resistance during ACh ejection versus nAChR depolarization amplitude. **H.** Example spontaneously active DAergic axon trace showing the change in AP height during control current injection train (black) and for a train in combination with ACh pressure ejection (green). **I.** Change in AP height from changes in axonal resting membrane potential (RMP) for control and ACh pressure ejection. *p<0.05, **p<0.01, ***p<0.001.

One possible mechanism for the nAChR inhibition of burst evoked DA release could emerge from the nAChR-mediated axon depolarizations, which may increase sodium channel inactivation and reduce axonal spiking (Zhang et al., 2025). A similar mechanism was proposed to reduce axonal excitability following activation of axonal GABAa receptors [9]. However, nAChR-mediated membrane depolarization may also favor axonal AP formation [24, 25]. To distinguish these two possibilities, we tested the effect of ACh on evoked firing in DAergic axons (Figures 3A and 3B). To assess spiking responses, we obtained perforated-patch recordings from DAergic axons in the medial fiber bundle (MFB) and evoked APs using a 50 Hz train of 10 current steps (1 ms duration) of increasing amplitude (+5 pA per sweep). We performed these experiments first under control conditions and then following a puff of 300 μM ACh (Figure 3C).

We found that puffing ACh resulted in a steady depolarization of the average axonal membrane voltage from −58.25 ± 1.53 mV to −55.69 ± 1.78 mV (n=15), and which reversed near 0 mV, as expected for neuronal nAChRs (Vernino et al., 1992) (Supplemental Figure 2). To analyze the effect of this on axonal spiking, we grouped the data into 3 categories based on the strength of the injected current relative the rheobase current for each recorded axon (*I*_Rh_; avg *I*_Rh_ = 135 +/− 21.5 pA) - low (*I*_Rh_ to *I*_Rh_ +10 pA), medium (*I*_Rh_ +15 to *I*_Rh_ +25 pA) and high (above *I*_Rh_+30 pA) current injection groups. We found a significant reduction in the likelihood of AP firing later in the burst train than at the start for all groups (F(9)=18.9, p<0.0001; Figure 3D, left). This effect was similar during nAChR depolarization (F(9)=20.29, p<0.0001; Figure 3D, right). The reduction in axonal excitability over the course of the burst could arise from an increase in potassium conductance, a decrease in sodium channel availability, or some combination. If sodium channel availability decreases throughout the burst, then the time to AP onset (peak of the first AP derivative) should increase concurrently. Consistent with this, the time to onset increased throughout the train from 1.68 ± 0.07 ms on pulse 1 to 2.1 ± 0.1 ms by pulse 10. This effect was similar during nAChR depolarization. Interestingly, the average time to peak was slightly shorter with nAChR activation suggesting an overall increase in excitability (ctrl: 1.90 ± 0.04 ms, ACh: 1.86 ± 0.04 ms, main effect of ACh: F(1,10)=5.45, p=0.04, n=11).

We next examined whether nAChR activation affected the likelihood of evoking an axonal AP. Because there is an intrinsic reduction in axonal excitability over the course of a burst, we examined successes/failures in control or during nAChR activation for the first 5 steps in the train. We found a significantly increased likelihood of AP initiation for all current steps from *I*_Rh_ to +50 pA (main effect of ACh: F(1, 428) = 11.95, p=0.0006; Figure 3F, left). Because current step amplitude and numbers were consistent between control and nAChR activation, we also counted the total numbers of action potentials for each current step for step amplitudes between *I*_Rh_ and +30 pA. We found that nAChR activation increased the number of APs throughout the train (control: 887 APs, ACh: 1005 APs out of 1930 current steps, Figure 3F, right).

We next analyzed the relationship between resting membrane potential and axonal spike height (Figure 3H and 3I). We found that activation of nAChRs had no effect on the fitted linear relationship between axonal spike height and resting membrane potential (slope, control, −0.031; ACh: −0.029; F(1, 278)=0.05, p=0.82). However, the Y-intercept was lower with nAChR activation than in control (y-intercept, control: 1.0, ACh: 0.96; F(1,278)=23.6, p<0.0001). Combined with the earlier result showing no detectable change in the axonal input resistance following nAChR activation (Figure 3G), this results suggests a subthreshold nAChR depolarization reduces spike peak, likely from sodium channel inactivation (Figure 3H and 3I).

Together, these results show that nAChR-mediated depolarizations increase the likelihood of local AP initiation. In addition, DAergic axons become less excitable during a 50 Hz AP train. Lastly, nAChR depolarization leads to a small but measurable decrease in the amplitude of propagating APs, which likely results from sodium channel inactivation rather than shunting.

### nAChRs inhibit DAergic axonal excitability as a consequence of robust excitation

**Figure 4.**
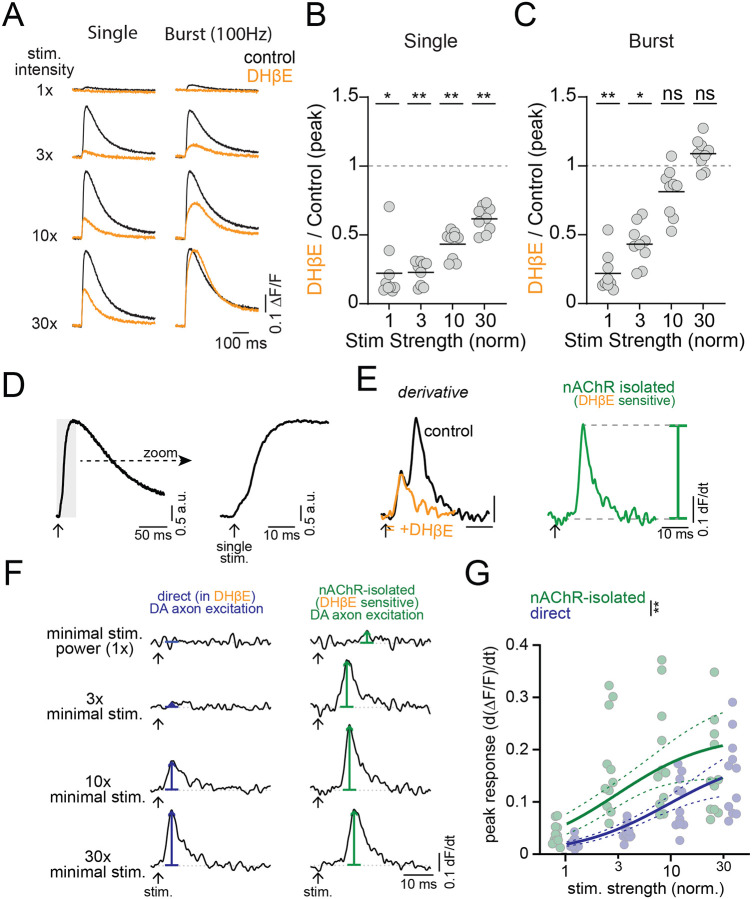
Weak stimulations produce nAChR excitation while strong stimulations produce nAChR inhibition **A.** Example traces for single and burst (5 stim at 100 Hz) protocols in control aCSF (black) and after nAChR inhibition with DHβE (1 μM, orange), across stimulation intensities. **B.** Inhibition of single-stimulus peak jGCaMP response by DHβE as a function of intensity. **C.** Inhibition of burst peak jGCaMP response by DHβE as a function of intensity. N=9 slices **D-E.** Analysis workflow for isolating direct and nAChR-mediated components from compound jGCaMP8f photodiode signals. **F.** Example differentiated jGCaMP8f traces showing direct DAergic axon activation (left, blue) and nAChR-mediated activation (right, green) across increasing stimulation intensities (top = minimal intensity). **G.** Peak differentiated jGCaMP8f signals for direct (blue) and nAChR-mediated (green) excitation plotted against normalized stimulation strength. Dashed lines on curve fits are 95% confidence bands. *p<0.05, **p<0.01, ns: p>0.05. Experiments performed with D2R, GABA-B, and mAChR antagonists.

*In vivo*, CIN activity within the local striatal network gives rise to varying levels of ACh release from tonic or burst activity (Aosaki et al., 1995; Wilson et al., 1990), resulting in a range of nAChR activation on dopaminergic axons. To test the sensitivity of Ca^2+^ responses in DAergic axons across a range of nAChR activation intensities, we imaged axonal Ca^2+^ signals while systematically varying electrical stimulation strength and constructed stimulation-response curves (see methods). We then repeated these experiments in the presence of DHβE to test the contribution of nAChRs to Ca^2+^ signals at different stimulation intensities. We found that single stimulus-evoked Ca^2+^ signals were reduced across all stimulation intensities by DHβE application (control-DHβE peak dF/F, stim 1x, t(8)=3.8, p=0.02; stim 3x, t(8)= 4.9, p=0.005; stim 10x, t(8)=4.6, p=0.007; stim 30x, t(8)=4.5, p=0.008), consistent with previous reports that nAChRs potentiate DA release (Figure 4A and 4B). Similarly, burst-evoked Ca^2+^ signals in response to low-intensity stimulation were significantly reduced by DHβE. However, the burst responses to strong stimulation were unaffected by DHβE (Figure 4A and 4C; control-DHβE peak dF/F, stim 1x, t(8)=4.8, p=0.006; stim 3x, t(8)=4.3, p=0.01; stim 10x, t(8)=2.5, p=0.14; stim 30x, t(8)=1.6, p=0.47).

Our results suggest that the interaction between CINs and nAChRs on DA axons depends on the intensity of the cholinergic stimulus, particularly during burst stimuli. To better understand this interaction, we distinguished Ca^2+^ influx due to nAChR excitation from that due to direct DAergic axon stimulation by differentiating the axonal Ca^2+^ signals (Brill-Weil et al., 2025) (Figure 4D and 4E). The differentiated Ca^2+^ signal was biphasic, consisting of an early peak followed by a second larger peak that was delayed in its timing. Following application of DHβE, the late second peak was abolished, consistent with the conclusion that it originates from nAChR activation (Brill-Weil et al., 2025). Plotting the differentiated Ca^2+^ signals for nAChR and direct DAergic axon evoked components against stimulation strength, we found that the half maximal response of the nAChR excitation occurred at a lower intensity than for direct DAergic axon stimulation (Kd F-test: 1,84) = 9.1; p = 0.003; Figure 4F and 4G). This observation is consistent with our findings of the undifferentiated signals, which show that nAChR-evoked signals dominate at weak to moderate stimulation strengths. Thus, CIN-mediated Ca^2+^ signals in DAergic axons are more sensitive to stimulation than the direct DAergic axon component, supporting the idea that DAergic axons are primarily excited by nAChRs over a broad range of activation intensities. Burst-evoked DA release is most prominent at the lowest stimulation intensities, whereas with increasing stimulation strength, nAChRs lead to little further effect of DAergic axonal excitability or may even suppress responses to burst stimulation.

### High-frequency burst spiking in DAergic axons induces refractory inhibition.

**Figure 5.**
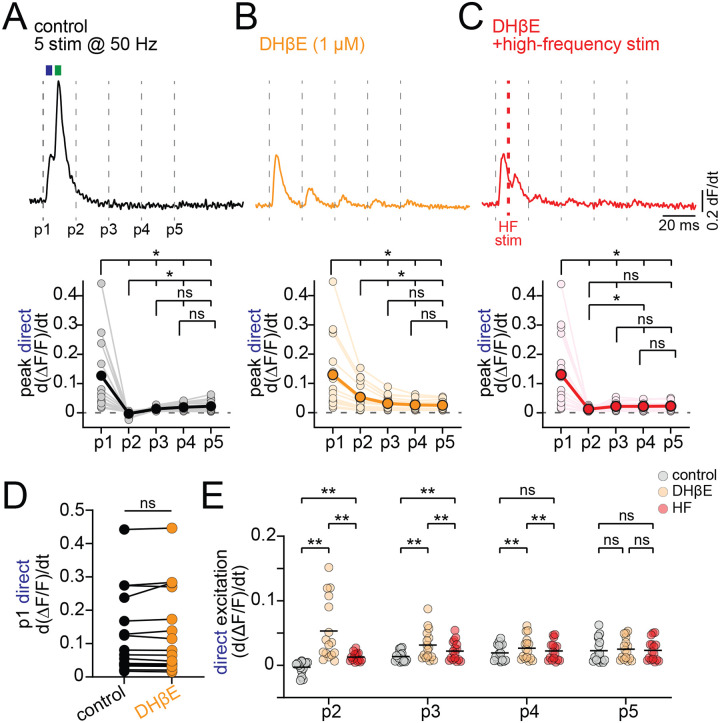
High-frequency stimulation reduces DAergic axonal excitation. **A.** (Top) Differentiated jGCaMP8f signal during 50 Hz DMS stimulation in control aCSF; dashed lines indicate individual pulses (P1–P5). Blue and green rectangles highlight direct and nAChR-mediated excitation, respectively. (Bottom) Group data for peak direct excitation across all 5 pulses. **B.** (Top) Example trace in DHβE; (Bottom) group data for peak direct excitation across 5 pulses. **C.** (Top) Example trace in DHβE with added electrical stimulation 6.8 ms after P1; (Bottom) group data for peak direct excitation across 5 pulses. **D.** Peak direct DAergic axon excitation (differentiated) for P1 in control and DHβE. **E.** Peak direct excitation for P2–P5 in control (grey), DHβE (orange), and DHβE with added high-frequency stimulation (red). *p<0.05, **p<0.01, ns > 0.05. Experiments performed in D2R, GABA-B, and mAChR antagonists.

We showed that Ca2+ signal evoked by high intensity nAChR activation results are enhanced following application of DHβE, suggesting that nAChRs can also play a role in reducing DAergic axon excitability. To test this, we recorded Ca2+ signals while stimulating high frequency bursts (5 stimulations at 50 Hz) in the DMS (Figure 5A). We differentiated the signals to separately analyze the direct and nAChR components. As reported earlier (Figure 4E), the differentiated Ca2+ signal immediately following P1 had a biphasic waveform, with an early initial direct DA and a late nAChR-mediated component. For pulses P2–5, however, there were no peaks observed suggesting that stimulation during the period following P1 was ineffective in evoking axonal Ca2+ signals (differentiated peak Ca2+, P1: 0.127 +/− 0.03; P2: −0.003 +/− 0.003; P3: 0.014 +/− 0.002; P4: 0.019 +/− 0.003; P5: 0.023 +/− 0.005; n=16; Figure 5A and 5E).

Following DHβE block of nAChRs, stimulation during pulses P2–4 was significantly more effective in evoking Ca2+ signals, which then showed measurable peak amplitudes (avg. differentiated Ca2+ signal amplitude for control vs DHβE; P2: 0.056, q(15)=5.55, p=0.004; P3: 0.018, q(15)=5.79, p=0.003, P4: 0.007, q(15)=5.77, p=0.003; Figure 5B and 5E), arguing that the inhibition was mediated by nAChR signaling. Of note, there was no significant difference between the differentiated peak signals of control and DHβE by the pulse 5 of the train, which occurs 80 ms after the initial stimulation (mean difference ctrl vs DHβE P5: 0.002, q(15)=2.53, p=0.21; Figure 5E), suggesting the initial mechanism of inhibition is limited to about 80 ms in duration. As a control for the effect of inhibiting nAChRs on total axonal excitability, we also quantified the amplitude of the direct DAergic axon excitation following the first pulse, before the nAChR signaling occurs, and found there was no significant change by DHβE (ctrl=0.13, DHβE =0.13, q(15)=1.07, p=0.74; Figure 5D).

We hypothesized that if the inhibition during pulse 2–5 reflects refractoriness caused by the nAChR-evoked high-frequency firing rather than a separate process, then it should be replicable in DHβE by using high-frequency stimulation to mimic the observed nAChR-dependent burst. To test this hypothesis, we modified the high-frequency burst stimulation protocol by inserting an additional pulse at 6.8 ms following pulse 1 (HF stim, Figure 5C). With the extra pulse, the HF burst stimulation frequency following P1 was 147 Hz, comparable to the average axonal burst frequency shown in Figure 2. We found that the HF stimulation significantly reduced the peak amplitude of differentiated Ca2+ signals for P2, P3 and P4 relative to DHβE (differentiated Ca2+ signal DHβE-HF; P2, q(15)=5.04, p=0.007; P3, q(15)=5.83, p=0.002; P4, q(15)=5.97, p=0.002; Figure 5E).

Thus, these data demonstrate that strong activation of nAChR results in a high-frequency burst of APs in DAergic axons that can lead to reduced axonal excitability for a period lasting 60–80 ms. Specifically, the period of inactivity likely reflects axon refractoriness that results from a high-frequency burst of AP firing caused by the activation of nAChRs.

## DISCUSSION

Here, by testing across a large range of stimulation intensities, we reveal new mechanisms by which nAChR signaling in DAergic axons can both positively and negatively modulate excitability in the DMS. While imaging jGCaMP8f, we show that axonal nAChR input potentiates Ca2+ signaling in DAergic axons at weak and moderate stimulation strengths. When recording from DAergic axons, we show that nAChR signaling can evoke high-frequency bursts of APs in the DMS, and we show that the subthreshold depolarization produced by nAChRs enhances AP initiation. These findings support the role of nAChRs as excitatory receptors in DAergic axons. We also find that strong stimulations can produce exaggerated periods of excitation followed by a reduction in axonal excitability, an outcome that also occurs after an interval of low-frequency tonic activity. This inhibitory pause is due to a refractory period of inhibition that is a consequence of the preceding excitation, and which reduces further spike initiation in the distal axon for up to 80 ms. Thus, these findings reveal a predominantly excitatory function of CIN influence over DAergic axonal excitability over most stimulation intensities, while also demonstrating that strong excitation leads to reduced excitability because of AP bursting.

### nAChR subthreshold signaling is mainly excitatory

The evidence that nAChR signaling in DAergic axons is excitatory has three main components. First, nAChR signaling in DAergic axons produces robust AP firing, including a novel form of bursting we describe above. Second, controlled application of ACh produces a subthreshold nAChR depolarization that increases the likelihood of AP initiation. Finally, for all but the strongest intensity stimulations, nAChR activity boosts Ca2+ signaling in DAergic axons during both single and burst protocols.

Striatal CINs spontaneously fire action potentials at low rates. This tonic level of activity produces moderate activation of DAergic axons (Kramer et al., 2022), compared to coordinated phasic bursting (Threlfell et al., 2012). Here we show that weak to moderate intensity stimulation produces large nAChR-dependent excitation for single and burst stimulation patterns. However, strong stimulation leads to nAChR-mediated excitation followed by a period of inhibition. We further show that the excitation-inhibition response is a two-part event that involves initial high-frequency action potential firing followed by a brief refractory period. AP bursts initiated in a DAergic axon also occur on top of a subthreshold depolarization envelope, which we show increases excitability, likely by bringing the axon closer to spike threshold. Because CINs are tonically active at a rate between 4 and 10 Hz, there is consistent nAChR depolarizations in DAergic axons (Kramer et al., 2022) that are more common than the occasional action potential, which is also more common than bursts of action potentials (Figure 2). We therefore suggest that enhancing excitability is the primary consequence of phasic nAChR signaling in DAergic axons.

However, the functional outcome of an axonal subthreshold depolarization is not as predictable as one in the somatodendritic compartment, and can sometimes lead to reduced excitability (Zbili & Debanne, 2019). Presynaptic inhibition was first described in primary sensory axons, where primary afferent depolarizations (PADs) mediated by GABAa receptor activation were found to reduce neurotransmitter release (Eccles et al., 1961), a process that predominantly involves GABAa shunting inhibition (Cattaert & El Manira, 1999). On DAergic axons, GABAa receptors are similarly depolarizing, but instead of being excitatory like nAChRs, they inhibit DAergic axonal excitability and reduce DA release (Kramer et al., 2020). This divergence in function likely stems from differences in the mechanisms of action. GABAa receptors on the DAergic axons mainly act through a tonic shunting inhibition, while this effect is minimal for nAChRs which mainly act through phasic depolarizations. Interestingly, phasic axonal GABAa depolarizations in the cerebellum (Pugh & Jahr, 2011) can potentiate synaptic transmission, and in certain axons of the spinal cord they can even enhance AP propagation past branch points (Hari et al., 2022), highlighting the complex nature of modulating excitability in axons.

Because GABAa and nACh receptors both produce depolarizations in DAergic axons, they also both produce a reduction in AP height. In recordings from DAergic axons of the MFB, we show that prolonged nAChR-dependent depolarization reduces peak AP amplitude of propagating spikes, likely because of a similar increased Na+ channel inactivation as with GABAa receptor depolarization. The amplitude of the axonal depolarization partly determines its excitatory or inhibitory function. While the pressure ejection-evoked nAChR depolarization shown in Figure 3 was comparable in amplitude to spontaneous nAChR events that we previously reported in DA terminals (Kramer et al., 2022), the depolarizations observed during the burst envelope were larger, ranging from 4–10 mV. Thus, the depolarization during the burst envelope would be expected to even further reduce AP height, which is consistent with our observations (see Figure 3). This increased effect on AP height may produce a large enough inhibition to suppress neurotransmitter release synergistically with the burst-mediated refractory period, potentially explaining the recently reported nAChR-mediated suppression of DA release (Zhang et al., 2025).

### *In vivo* modulation of DAergic axons by CINs is likely excitatory

Here we report that low to moderate stimulation intensities mainly excite DAeric axons, while only the strong stimulations produce a net period of refractory inhibition. *In vivo*, CINs are spontaneously active, with tonic firing rates between 4 and 10 Hz, and can fire bursts of APs around 20 Hz (Aosaki et al., 1995; Duhne et al., 2024; Wilson et al., 1990), which are often faster than measurements *in vitro* that are often reported between 1 and 4 Hz (Bennett & Wilson, 1999; Dorst et al., 2020). Because ACh release from CINs is depressing (Mamaligas & Ford, 2016), we expect that phasic ACh release onto DAergic axons would be depressed *in vivo* relative to the present experiments. Therefore, the level of ACh activation of nAChRs *in vivo* likely represents the low to moderate stimulation strengths reported here, which we predict would be a predominantly excitatory signal. In response to salient stimuli, CINs will fire synchronized bursts (Ravel et al., 1999, 2003; Raz et al., 1996), which could produce strong stimulation that would lead to bursting in DAergic axons and an ensuing period of refractory inhibition. Of note, we found that optogenetic activation of CINs produced AP trains in DAergic axons with significantly more spikes than we saw with electrical stimulation. Therefore, optogenetic activation may bias experimental results towards a strong stimulation outcome that includes prolonged refractory periods of inhibition.

### Inhibitory modulation of DAergic axons by nAChR signaling

In past work, nAChRs were proposed to act as a low-pass filter on DA release, enabling DA transmission at low frequencies while inhibiting DA release from high-frequency bursting at rates above 25 Hz (Rice & Cragg, 2004; Zhang & Sulzer, 2004). The cellular mechanism mediating this filtering has remained largely unresolved. Here, we show that nAChR activation is not a low-pass filter but rather can evoke high-frequency spiking, with instantaneous firing rates of ~120 spikes per second. However, this period of strong excitation and nAChR-mediated bursting occurs only during the onset of the nAChR depolarization, within the first 20 ms of nAChR signaling. Evidence for this comes from both direct voltage recordings and from population-level calcium derivatives. Following this short period of excitation there is a longer period of refractory inhibition that is a consequence of the intense excitation. Because we can mimic the nAChR refractory inhibition in the presence of nAChR antagonists with only high-frequency electrical stimulations (see Figure 5), we propose this inhibitory mechanism is not unique to nAChR signaling. Any period of strong excitation in DAergic axons would be expected to produce an ensuing period of inhibition, following the model of refractory inhibition first described by Hodgkin and Huxley (Hodgkin & Huxley, 1952).

### Burst frequency adaptation in DAergic axons

Burst frequency adaptation occurs in the cell bodies of ventral midbrain DANs, with slower inter-spike intervals towards the end of a train of APs (Shepard & Bunney, 1991). This adaptation suggests that channels are activated in the soma and dendrites of DANs during an AP burst that reduce excitability. However, it was unknown whether a similar mechanism occurred for APs initiated in DAergic axons. Here we show by evoking AP firing in DAergic axons that the excitability of the axonal membrane also decreases over the duration of a high frequency AP burst. The evidence for a reduction in excitability during a high-frequency burst comes from a reduction in AP initiation probability and a slowing of the time to AP onset over the course of a 10-pulse burst at 50 Hz. The mechanism of this adaptation is likely to be prolonged Na+ channel inactivation, which could occur through a slow-inactivation gate (Do & Bean, 2003) that is suggested to be present in the soma and dendrites of DANs (Ding et al., 2011). This period of refractory inhibition also resembles depolarization block, a process that has been well characterized in the somata of DANs (Blythe et al., 2009; Lammel et al., 2008; Richards et al., 1997; Tucker et al., 2012). It is also possible axonal potassium channels are activated over the duration of a burst that then decrease axonal excitability. One such channel is KCNQ4, which is present in DAN somata and dendrites, (Drion et al., 2010) and can modulate striatal DA release (Jensen et al., 2011), but which has not been characterized in DAergic axons.

### AP bursting initiated in the axons of dopaminergic neurons

DANs of the ventral midbrain have two main firing modes, tonic and bursting. Tonic firing is linked to movement behaviors and is thought to set a baseline level of DA release. By contrast, AP bursting of midbrain DANs is a phasic signal that drives DA-dependent learning in response to salient sensory stimuli (Hart et al., 2014; Kutlu et al., 2021; Schultz et al., 1997). The difference between the tonic DA signal and burst-mediated DA signal may convey information about the sensory experience. Interestingly, the burst firing we observed in DAergic axons occurs at faster frequencies than is typical for DAN somata in the SNc (Grace & Bunney, 1983; Otomo et al., 2020). Therefore, it is reasonable to hypothesize that the ultra-fast burst of APs evoked by CIN input onto DAergic axons may convey different information from somatic bursting. However, whether CINs evoke bursting in DAergic axons in vivo is unclear, and what’s more the behavioral conditions under which CINs produce any DA release remain mostly unresolved, leaving open unresolved questions about the relationship between CIN-mediated ACh signaling and DA release.

## METHOD

### Mice:

All animal handling and procedures were approved by the animal care and use committee (ACUC) for the National Institute of Neurological Disorders and Stroke (NINDS) at the National Institutes of Health. Mice of both sexes were used throughout the study. Mice that underwent viral injections were injected at postnatal day 21 or older and were used for ex vivo imaging 1–4 weeks after injection. DAT-Cre (RRID:IMSR_JAX:006660) and Ai9 (RRID:IMSR_JAX:007909) strains were used.

### Viral injections:

All stereotaxic injections were conducted on a Stoelting QSI (Cat#53311). Mice were maintained under anesthesia for the duration of the injection and allowed to recover from anesthesia on a warmed pad. The viruses used for this study were jGCaMP8f (AAV9-pGP-syn-FLEX-jGCaMP8f-WPRE titer: > 1× 1013, Addgene #162379) and cre-dependent tdTomato (AAV9-CAG-FLEX-tdTomato titer: > 1× 1012, plasmid: Addgene #28306). Viral aliquots were injected (0.1–0.5 μl) bilaterally into the SNc (X: ± 1.9 Y: −0.5 Z: −3.9) via a Hamilton syringe. At the end of the injection, the needle was raised at a rate of 0.1 to 0.2 mm per minute for 10 minutes before being removed.

### Tissue sectioning:

Experiments were performed on male and female adult mice of at least 6 weeks in age. Mice were anesthetized with isoflurane, decapitated, and brains rapidly extracted. Horizontal sections were cut at 330 μm thickness on a vibratome while immersed in warmed, modified, slicing ACSF containing (in mM) 198 glycerol, 2.5 KCl, 1.2 NaH2PO4, 20 HEPES, 25 NaHCO3, 10 glucose, 10 MgCl2, 0.5 CaCl2. Cut sections were promptly removed from the slicing chamber and incubated for 30–60 minutes in a heated (34°C) chamber with holding solution containing (in mM) 92 NaCl, 30 NaHCO3, 1.2 NaH2PO4, 2.5 KCl, 35 glucose, 20 HEPES, 2 MgCl2, 2 CaCl2, 5 Na-ascorbate, 3 Na-pyruvate, and 2 thiourea. Slices were then stored at room temperature and used 30 min to 8 hours later. Following incubation, slices were moved to a heated (33–35°C) recording chamber that was continuously perfused with recording ACSF (in mM): 125 NaCl, 25 NaHCO3, 1.25 NaH2PO4, 3.5 KCl, 10 glucose, 1 MgCl2, 2 CaCl2. In experiments where there were drugs in the circulating ACSF, the slices were incubated in the recording solution for at least 15 minutes before recording began.

### Electrophysiology and functional imaging:

For imaging experiments, a white light LED (Thorlabs; SOLIS-3C) was used in combination with a EGFP (Chroma; 49002) filter set to visualize DAergic axons infected with jGCaMP8f. For visualizing the jGCaMP8f signals, a photodiode (New Focus) was mounted on the top port of the Olympus BX-51WI. Signals were using a Digidata 1550 (Molecular Devices) sampled at 50kHz. All electrical stimulation was delivered with tungsten bipolar electrodes (250 μm tip separation, MicroProbes) placed 100–200 μm from the imaging site in the DMS. Electrical stimulation was given using an Isoflex (A.M.P.I.) with amplitudes ranging from 0.1 to 3 V. For stimulation-response curves, a minimal stimulation intensity was experimentally determined for each replicate by reducing the stimulation intensity until there was no observable response. Then, the intensity was increased until a minimal response emerged, which was set as the “minimal” intensity (1). Stimulations were then increased as multiples of this normalized minimal intensity (3x, 10x, and 30x). Experiments in “iso. aCSF” were in the presence of GABAb (CGP-55845, 300 nM), mAChR (atropine, 100 nM), and D2R (sulpiride, 300 nM) inhibitors.

Perforated-patch recordings from striatal TdTomato+ axons were made using borosilicate pipettes (5–9 MΩ) filled with internal solution containing (in mM) 135 KCl, 10 NaCl, 2 MgCl2, 10 HEPES, 0.5 EGTA, 0.1 CaCl2, adjusted to a pH value of 7.43 with KOH, 278 mOsm. Pipette tips were back-filled with ~1 μL of clean internal. Pipettes were then filled with internal containing between 80 and 100 μg/mL gramicidin. Patch integrity was monitored by the addition of Alexa-488 to the gramicidin-containing internal. Whole-cell recordings from the TdTomato+ axons in the medial forebrain bundle were made using borosilicate pipettes (4–7 MΩ) filled with internal solution containing (in mM) 122 KMeSO3, 9 NaCl, 1.8 MgCl2, 4 Mg-ATP, 0.3 Na-GTP, 14 phosphocreatine, 9 HEPES, 0.45 EGTA, 0.09 CaCl2, adjusted to a pH value of 7.35 with KOH. All recordings were made with a MultiClamp 700B (Molecular Devices).

Pressure ejection of ACh was performed using borosilicate pipettes (2–4 MΩ). ACh (300 μM) was added to a modified external solution containing (in mM): 125 NaCl, 25 NaHCO3, 1.25 NaH2PO4, 3.5 KCl, 10 HEPES, 0.01 Alexa 488, final osmolarity 280–290 mOsm. This puffing solution was then spin filtered, loaded into a glass pipette, and lowered to within 30–50 μm of the axon using a micro-manipulator. The puffing solution was applied onto the axon with a short pressure ejection (100–250 msec in duration) using a PV 820 Pneumatic PicoPump (WPI).

The starting amplitude of the current injection protocol in Figure 4 was experimentally tuned for each axon to be close to rheobase (*I*_Rh_), and increased by 5 pA for each subsequent trial (Figure 4B). 10 current steps were performed with a 20 ms inter-stimulus interval. The protocol was continued until a 100% success rate of spiking was achieved. For analysis, only sweeps prior to the 100% successes of AP initiation were considered.

### Quantification and statisitcs

Analysis was conducted in Igor Pro (Wavemetrics) and statistical tests were performed in Prism 9 (GraphPad) and Igor Pro. Parametric data are reported as the mean while non-parametric are reported as the median. Error bars for non-parametric data are shown as standard deviation (s.d.), while those for parametric data are standard error of the mean (s.e.m.). Data in box-and-whisker plots (non-parametric) are showns as: the box line is at the median, the hinges of the box extend from the 25th to 75th percentiles, and the whiskers extend from the minimum to maximum in the dataset. T-tests were used for two-group comparisons, and ANOVA tests were used when comparing more than two groups followed by a Bonferroni post-hoc test for analysis of multiple comparisons. Cumulative distributions were compared with a Kolmogorov-Smirnov test. Statistics are shown t(df)=t_val where df indicates the degrees of freedom and Y indicates the calculated test statistic.

#### Action potential analysis

To quantify APs, the derivative was taken of each trace in the recording and the stimulation artifacts were blanked. The derivative traces were analyzed in Igor Pro using NeuroMatic (Rothman & Silver, 2018). A cutoff value was set to identify AP derivatives that were at least 2 standard deviations above the baseline noise. Derivatives were then manually confirmed to be APs. The AP success rate in Figure 4 was calculated across axons for each stimulation pulse (1 through 10) in control (black) and during ACh ejection (green).

#### Discrimination between direct and nicotinic components

For discrimination between the direct and nicotinic components of the evoked jGCaMP8f signal, the average waveform of at least 2 evoked Ca2+ events were taken under control conditions. The “direct component” time window was defined as the time between the onset (5% of peak) of the first peak and the onset of the second peak. This resulted in a region that was a 5.6 ms window beginning 1.1 ms after the stimulus. The analysis window for the nicotinic component was defined as a 20 ms window immediately following the direct component. This analysis is shown in Figure 4. The analysis starts with a raw jGCaMP8f signal, which is first differentiated. The application of DHβE defines the direct differentiated signal in DAergic axons. The direct differentiated signal is then subtracted from the control signal to produce an isolated nAChR-mediated differentiated signal.

#### Amplitude quantification of direct and nicotinic components

In order to quantify changes to the direct component of evoked jGCaMP8f signals, the first derivative of the raw signal was low pass filtered. The maximum value within the direct component analysis window was determined for every trace in each slice to create a time series. To quantify the nicotinic component for each slice, the average first derivative trace in DHβE was subtracted from each individual first derivative wave of the recording. This resulted in first derivative waves that contained only the nicotinic contribution to the jGCaMP8s signal. To make the analysis robust to noise and account for any changes in the direct component amplitude, each individual trace was then baselined to the second half (2.8 msec) of the direct component window. The peak value was taken for every wave within the analysis window for the nicotinic component, as defined above. To account for baseline noise in the first derivative photometry traces, the average noise was determined for each slice using a 5.6 msec window immediately before the stimulus. This average value from all waves in each slice was subtracted from the quantification of the peak nicotinic signal. Finally, both the quantifications of direct and adjusted nicotinic components for each slice were normalized to control conditions and averaged across slices. Quantification of drug effects was done by averaging the final 5 minutes of the control or wash-in periods.

## Supplementary Material

1
