# Supplementary material for: High-frequency axonal bursts mediate bidirectional modulation of dopamine signaling by nicotinic receptors
Source: bioRxiv. 2026 Feb 3:2025.12.19.695584. Originally published 2025 Dec 21. Preprint. [Version 2] doi: 10.64898/2025.12.19.695584 (PMC12724510; doi:10.64898/2025.12.19.695584)
Supplement: Supplement 1 [file NIHPP2025.12.19.695584v2-supplement-1.pdf]

bioRxiv preprint doi: https://doi.org/10.64898/2025.12.19.695584; this version posted February 3, 2026. The copyright holder for this preprint (which was not certified by peer review) is the author/funder, who has granted bioRxiv a license to display the preprint in perpetuity. It is made available under aCC-BY 4.0 International license.

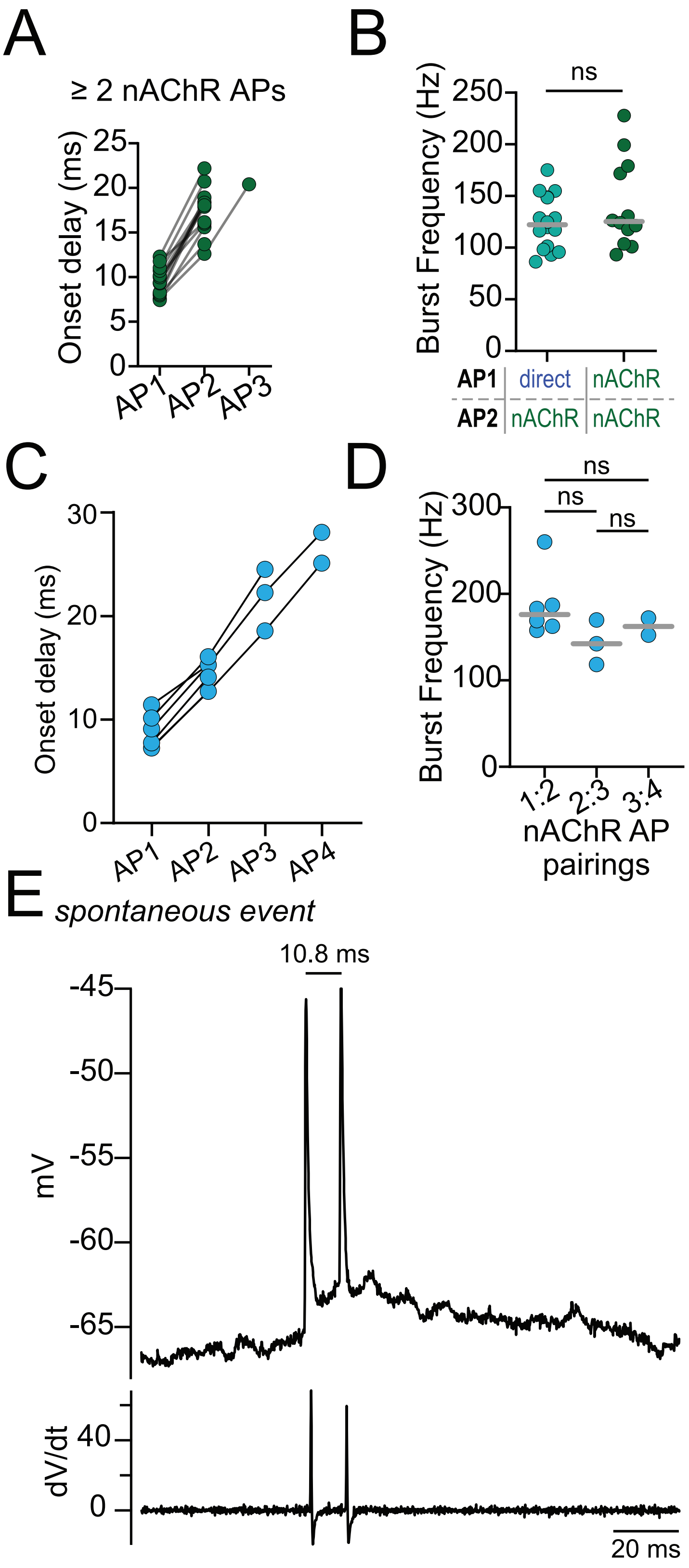

Supplemental Figure 1. Further characterization of bursting in dopaminergic axons

A. Action potential onset delay following a single electrical stimulation for nAChR-mediated spikes in records with > 2 events.
B. No significant difference in the instantaneous frequencies between pairs of APs that combine a direct and nAChR-mediated spike, or two nAChR-mediated spikes.
C. Action potential onset delay following a single LED light pulse for nAChR-mediated spikes in records with > 2 events.
D. No significant difference in the instantaneous frequency between pairs of APs throughout the LED-evoked burst train.
E. Example recording of a spontaneously generated high-frequency burst of APs recorded in a DAergic axon on the DMS.

bioRxiv preprint doi: https://doi.org/10.64898/2025.12.19.695584; this version posted February 3, 2026. The copyright holder for this preprint (which was not certified by peer review) is the author/funder, who has granted bioRxiv a license to display the preprint in perpetuity. It is made available under aCC-BY 4.0 International license.

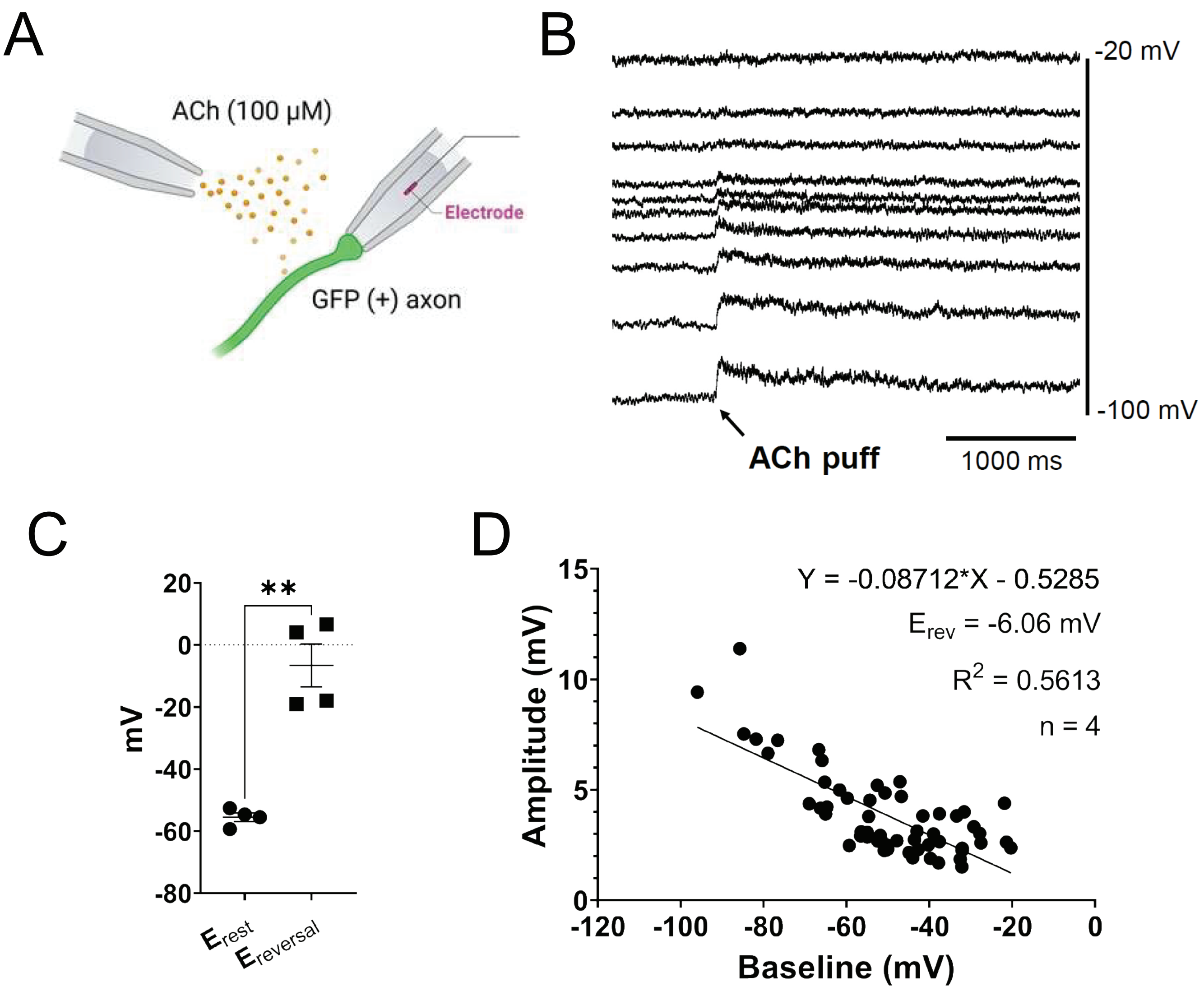

Supplemental Figure 2. nAChR potentials reverse near 0 mV in dopaminergic axons

A. Experimental setup: DAergic axons in the MFB were recorded using perforated patch recordings from MFB axons; nAChRs activated by local ACh ejection.
B. Example recording showing an ACh-mediated depolarization at different resting membrane potentials.
C. Combined data showing a significantly depolarized reversal potential for nAChRs relative to the axonal resting membrane potential.
D. Combined data showing the calculation of a reversal potential using linear estimation.
** $p<0.01$
