# Supplementary material for: Excitatory nicotinic signaling drives action potential bursting in dopaminergic axons
Source: bioRxiv. 2025 Dec 21:2025.12.19.695584. Preprint. [Version 1] doi: 10.64898/2025.12.19.695584 (PMC12724510; doi:10.64898/2025.12.19.695584)
Supplement: Supplementary file 1 [file NIHPP2025.12.19.695584V1-supplement-1.pdf]

bioRxiv preprint doi: https://doi.org/10.64898/2025.12.19.695584; this version posted December 21, 2025. The copyright holder for this preprint (which was not certified by peer review) is the author/funder, who has granted bioRxiv a license to display the preprint in perpetuity. It is made available under aCC-BY 4.0 International license.

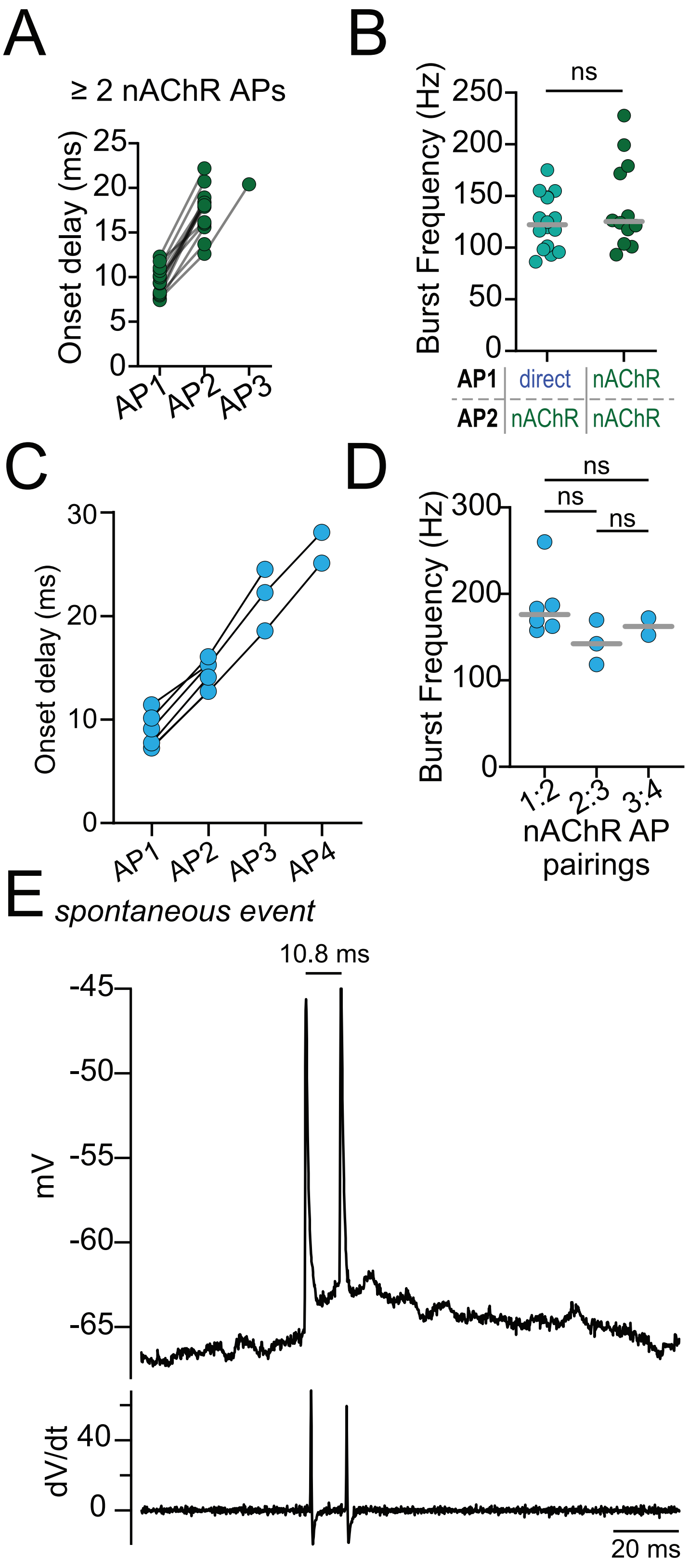

Supplemental Figure 1. Further characterization of bursting in DAergic axons

A. Action potential onset delay following a single electrical stimulation for nAChR-mediated spikes in records with > 2 events.
B. No significant difference in the instantaneous frequencies between pairs of APs that combine a direct and nAChR-mediated spike, or two nAChR-mediated spikes.
C. Action potential onset delay following a single LED light pulse for nAChR-mediated spikes in records with > 2 events.
D. No significant difference in the instantaneous frequency between pairs of APs throughout the LED-evoked burst train.
E. Example recording of a spontaneously generated high-frequency burst of APs recorded in a DAergic axon on the DMS.

bioRxiv preprint doi: https://doi.org/10.64898/2025.12.19.695584; this version posted December 21, 2025. The copyright holder for this preprint (which was not certified by peer review) is the author/funder, who has granted bioRxiv a license to display the preprint in perpetuity. It is made available under aCC-BY 4.0 International license.

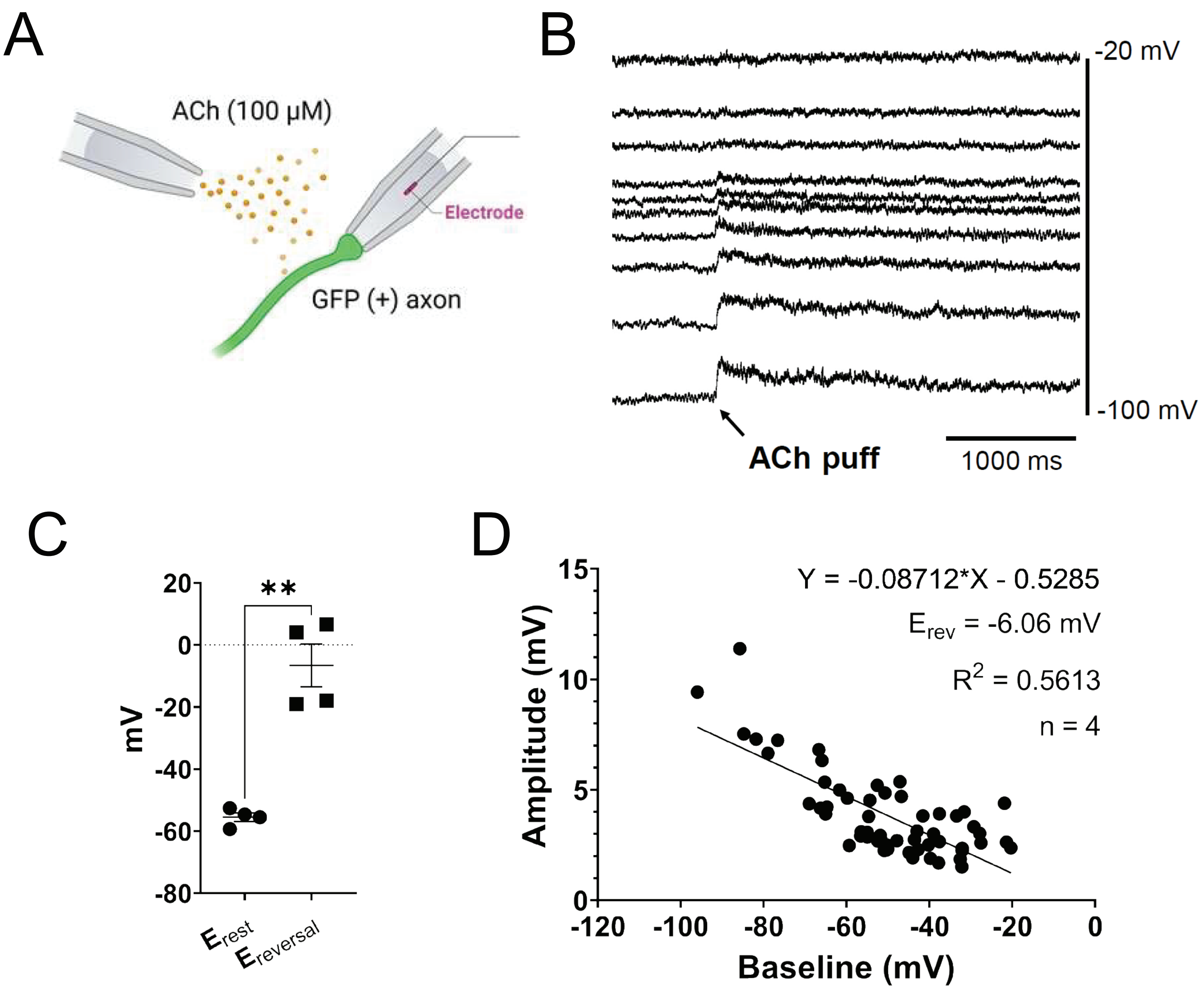

Supplemental Figure 2. nAChR potentials reverse near 0 mV in dopaminergic axons

A. Experimental setup: DAergic axons in the MFB were recorded using perforated patch recordings from MFB axons; nAChRs activated by local ACh ejection.

B. Example recording showing an ACh-mediated depolarization at different resting membrane potentials.

C. Combined data showing a significantly depolarized reversal potential for nAChRs relative to the axonal resting membrane potential.

D. Combined data showing the calculation of a reversal potential using linear estimation.

** $p<0.01$
